# Piezoelectric Transducer-Based Structural Health Monitoring for Aircraft Applications

**DOI:** 10.3390/s19030545

**Published:** 2019-01-28

**Authors:** Xinlin Qing, Wenzhuo Li, Yishou Wang, Hu Sun

**Affiliations:** School of Aerospace Engineering, Xiamen University, Xiamen 361005, China; liwenzhuo@stu.xmu.edu.cn (W.L.); wangys@xmu.edu.cn (Y.W.)

**Keywords:** structural health monitoring, piezoelectric transducer, sensor network, damage detection, aircraft

## Abstract

Structural health monitoring (SHM) is being widely evaluated by the aerospace industry as a method to improve the safety and reliability of aircraft structures and also reduce operational cost. Built-in sensor networks on an aircraft structure can provide crucial information regarding the condition, damage state and/or service environment of the structure. Among the various types of transducers used for SHM, piezoelectric materials are widely used because they can be employed as either actuators or sensors due to their piezoelectric effect and vice versa. This paper provides a brief overview of piezoelectric transducer-based SHM system technology developed for aircraft applications in the past two decades. The requirements for practical implementation and use of structural health monitoring systems in aircraft application are then introduced. State-of-the-art techniques for solving some practical issues, such as sensor network integration, scalability to large structures, reliability and effect of environmental conditions, robust damage detection and quantification are discussed. Development trend of SHM technology is also discussed.

## 1. Introduction

For metallic or composite aircraft structures, in-service conditions and failure modes are generally complex and may not be accurately predicted. Because of this, the aerospace industry typically uses conservative time-based or usage-based scheduled maintenance practices that are overly time-consuming, labor-intensive, and very expensive. Furthermore, as structures age, maintenance service frequency and costs increase while performance and availability decrease. One possible method of ensuring structural safety is to inspect structures frequently and keep abreast of their structural condition. The use of condition-based maintenance coupled with continuous on-line structural integrity monitoring could significantly reduce the cost of inspection.

Structural Health Monitoring (SHM) is perceived as a significant method for determining the integrity of structures involving the use of multidisciplinary fields including sensors, materials, signal processing, system integration and signal interpretation [1,2,3]. The aim of SHM technology is not simply to detect structural failures, but also to provide an early indication of physical damage. The early warnings provided by an SHM system can then be used to define remedial strategies before the structural damage leads to failure. It shows great promise of being embraced by the aerospace industry as capable of monitoring the structural condition throughout its service lifetime [4,5]. The general perspectives of aircraft company on SHM include increasing safety and reliability, reducing maintenance cost, saving structure weight, and reducing operation cost. The key features of SHM technology comparing to traditional nondestructive testing (NDT) technology are listed in Table 1.

During the past two decades, different SHM system technologies, including both passive and active monitoring, have been developed through the use of built-in distributed sensor network integrated with metallic and composite aircraft structures [6,7,8,9]. There have been a number of prototypes and laboratory demonstrations of SHM systems that have been presented and displayed at meetings, conferences, and workshops. As shown in Table 2, many sensors can be used for SHM, such as optical fibers, piezoelectric materials (such as piezoelectric lead zirconate titanate (PZT)), nanomaterials, air/vacuum galleries and Eddy current foil sensors [10,11,12,13]. Among these various types of transducers, piezoelectric materials are being widely used for SHM because they can be used as either actuators or sensors due to their piezoelectric effect and vice versa.

While the SHM technology has shown great promise in the laboratory, the transition into field applications have been pretty slow. While most of major civil aircraft OEMs, such as Airbus, Boeing and COMAC, have been paying close attention to it, and evaluating it in the real world, none fully succeeded to incorporate it within their civil aircrafts yet. This is due to a number of factors including [14]:

(1) Optimized sensor layout: Most SHM systems require an expert with years of experience to know where and how to install sensors for optimal damage detection for every new application.
(2) Self-diagnostics: Most SHM systems do not have built-in self-diagnostics and thus cannot distinguish between damage to the structure and damage to the sensors themselves.
(3) Environmental compensation: Most SHM systems are overly sensitive to environmental changes and do not have effective compensation techniques to allow practical field use.
(4) Probability of detection: Most SHM systems cannot easily provide quantifiable specifications for resolution or probability of detection (POD) for every new application or sensor configuration.
(5) Damage quantification: Most SHM systems do not output quantitative damage sizes with associated uncertainty values.
(6) Airworthiness compliance: the SHM technology needs to meet not only the intended functions for a specific application, but also the requirements of airworthiness compliance

A complete, robust SHM system is one that combines the essential elements needed for implementation including state-of-the-art techniques to optimize sensor placement, perform self-diagnostics, compensate for the influence of environmental variation, generate POD curves and output quantitative damage sizes.

This paper first provides a brief overview of piezoelectric transducer-based SHM technology developed in the past two decades. The piezoelectric transducer-based SHM systems including sensor network and diagnostic hardware with principles of monitoring, identification algorithms of damage and impact are summarized. The requirements for practical implementation and use of SHM systems in aircraft application including the design principles of SHM systems and some key issues are then introduced. State-of-the-art techniques for solving some practical issues, such as sensor network integration, scalability to large structures, reliability and effect of environmental conditions, robust damage detection and quantification are discussed. Development trends of SHM technology are also discussed in this paper.

## 2. Piezoelectric Transducer Based SHM System

**Figure 1:**
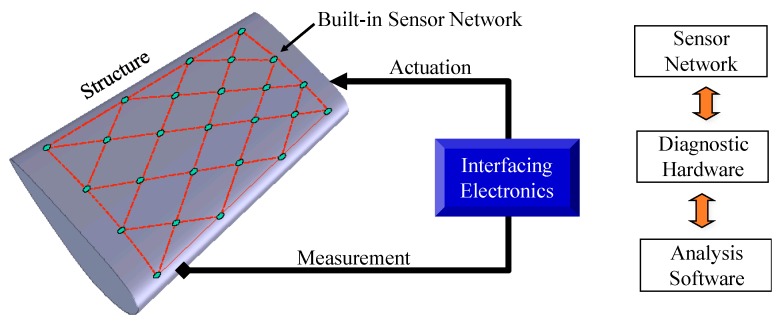
Composition of piezoelectric sensor network based SHM system.

A typical piezoelectric transducer based SHM system consists of a sensor network mounted on or embedded into a host structure, portable diagnostic hardware, and data analysis software, as shown in Figure 1.

### 2.1. Sensor Network

Sensors that transform the parameters of structure state, such as damage, load and temperature, into the corresponding sensing signals are the basis of SHM. They are usually mounted on the surface of a structure or embedded into the structure in the form of a network. The sensor network plays an important role in the performance of the SHM system. The ability of sensors and actuators in the network to communicate with each other defines the intelligence of the system. The type, location, and number of sensors and actuators critically affect the sensitivity and performance of the SHM system. As the number of sensors increases, the integration of such a sensor network with a structure can be very challenging or become impractical. The SMART Layer technology originally developed at Stanford University offers a simple and efficient way to integrate a large PZT sensor network onto a structure with high reliability [15,16]. Once it is mounted, the sensor network can be used to collect health monitoring data throughout the service life of the structure.

**Figure 2:**
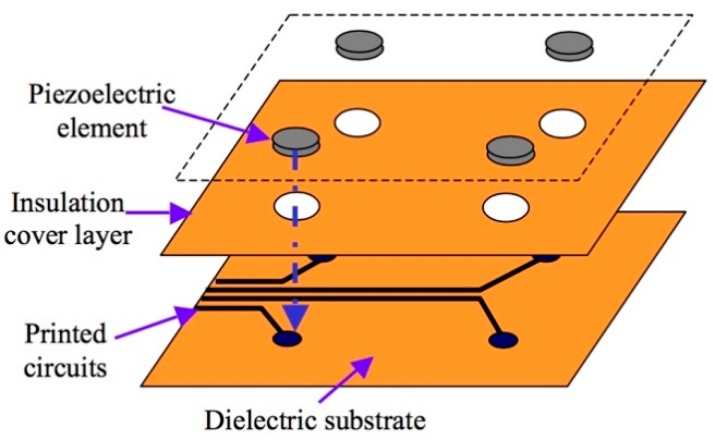
Basic configuration of sensor layer [17].

The layer is made of a thin dielectric film with an embedded network of distributed PZTs that can be used as either actuators or sensors, as shown in Figure 2. It utilizes a layered construction: a circuit layer, an insulation layer, and a sensor layer [17]. The novelty of the layer lies in its networking capabilities with any type of sensor enhancing its monitoring capabilities and eliminating the need to place each type of sensor individually on the structure [15].

**Figure 3:**
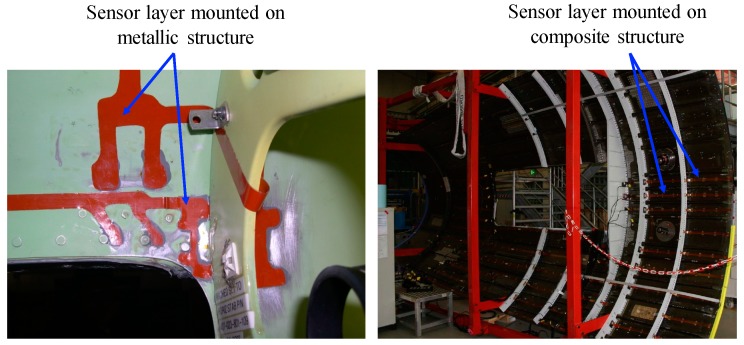
Sensor layers mounted on the surfaces of metallic and composite structures.

**Figure 4:**
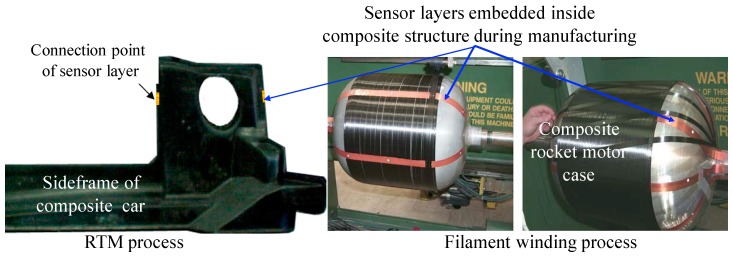
Sensor layers embedded inside composite structure during different manufacturing process (adapted from [18,19]).

The characteristic features of the layer include: (1) ease of installation, (2) adaptability to any structure with complex geometry, (3) use of an area sensing network, (4) actuation and sensing capabilities, (5) signal consistency and sensor reliability, and (6) shielding to reduce EM noise. The layer can either be surface-mounted on existing structures or embedded inside composite structures during fabrication thereby providing a built-in nondestructive assessment of the structure states. Figure 3 shows the sensor layers mounted on the surfaces of hot spot areas of a helicopter and prototype of composite fuselage. Figure 4 shows the sensor layers embedded inside a side-frame of composite car and a filament bottle during manufacturing [16,18,19].

Similar to SMART Layer, another novel lightweight diagnostic film with sensors/actuators and a multiple-path wiring option using inkjet printing was recently developed. The diagnostic film allows for systematic, accurate, and repeatable sensor placement. Furthermore, the film is highly flexible and adaptable for placement on complex configurations. The diagnostic film offers significant weight reduction compared to other available technologies [20].

### 2.2. Diagnostic Hardware and Principles of Monitoring

Piezoelectric sensor networks integrated with a structure can be used for both active and passive sensing. In the active-sensing mode, both wave propagation and electromechanical impedance (EMI)-based SHM methods have been developed for damage detection. The passive SHM mode is used in real time to monitor impact events including both impact location and energy.

#### 2.2.1. Wave Propagation-Based SHM

**Figure 5:**
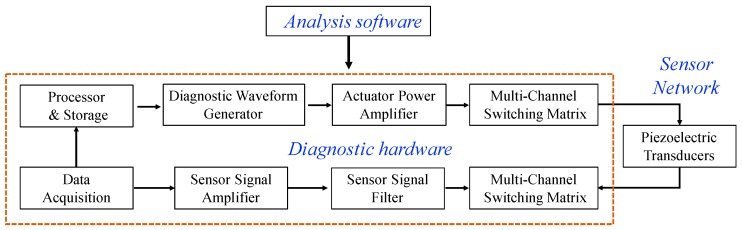
Diagram of the integrated active diagnostic hardware (adapted from [21]).

A typical active diagnostic hardware for wave propagation-based SHM has the built-in capability to generate a specific waveform for structural diagnostics, collect sensor data at a high sampling rate and resolution, and multi-channel capability to accommodate a network of piezoelectric elements. As shown in Figure 5, the components of hardware include a diagnostic waveform generator, an actuator power amplifier, a multi-channel switching matrix, a sensor signal filter and amplifier board, a sensor data acquisition board, and devices for data storage and processing [21]. In general, the diagnostic waveform output used to drive the piezoelectric actuators is a five-peak sine wave modulated by a cosine (Gaussian) envelope [15].

**Figure 6:**
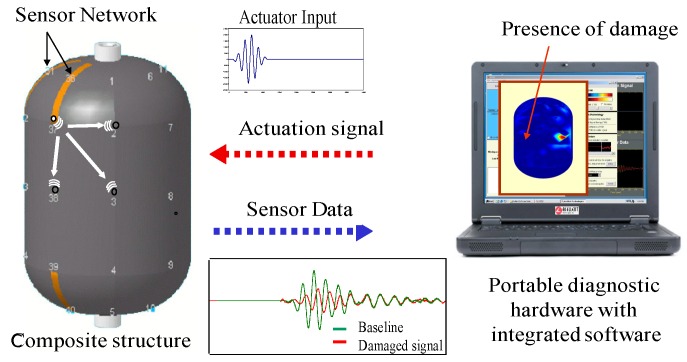
Principle of wave propagation-based SHM.

In the wave propagation-based SHM, guided waves, such as Lamb and Rayleigh waves, are most widely used for damage detection in metallic and composite structures. Guided waves used for damage detection are introduced into a structure at one point by a piezoelectric actuator and sensed by another piezoelectric sensor at a different position (pitch-catch), or the same piezoelectric element (pulse-echo), as shown in Figure 6.

A key advantage of using piezoelectric elements is that a larger area of the structure can be monitored with fewer transducers, which is vitally important for the monitoring of large-scale structures. Other sensors, like optical fiber-based types, can only scrutinize smaller, specific areas, thus leaving larger areas of a structure unmonitored.

#### 2.2.2. EMI-Based SHM

The electromechanical impedance (EMI) technique is another active SHM method promoted by piezoelectric transducers. In the EMI-based SHM, the electrical parameters of a piezoelectric transducer are measured to identify damage. The damage near the transducer causes a stiffness change and affects the structure’s resonant characteristics which will accordingly change the electrical impedance of transducer due to the electromechanical coupling. The diagnostic process of active SHM is usually based on the comparison of the current sensor responses with previously recorded sensor responses (baselines) from the undamaged structure. The differences between the two sets of signals are what contain the information about any existing damage or other anomalies. Since the EMI method was first proposed in 1993 by Liang [22], it has gained extensive attentions on the applications of damage detection in civil infrastructures, mechanical engineering and aerospace engineering [23,24]. A lot of work has been made in the theoretical models [25,26,27], 2D or 3D numerical modeling and simulation [28,29,30], and system development [31].

**Figure 7:**
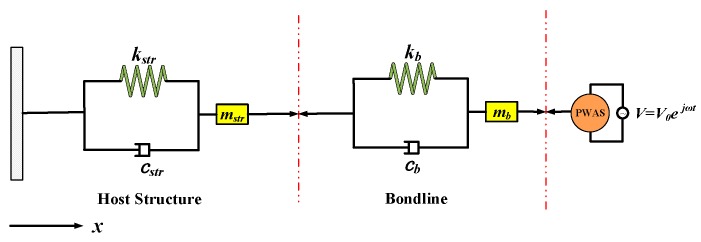
SMD equivalent model considering bondline.

**Figure 8:**
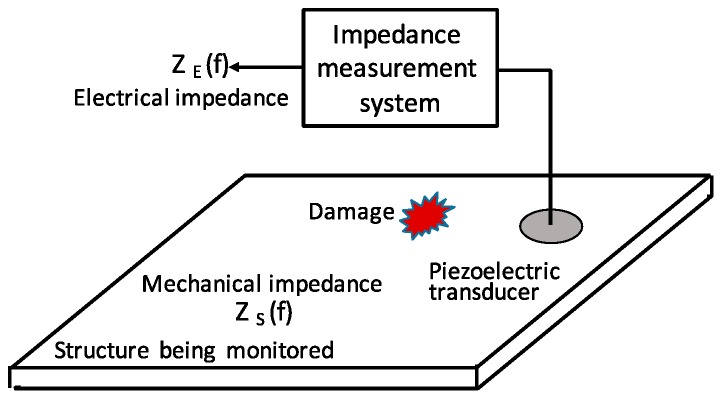
Principle of the EMI method.

Based on the previous study [22,32], the piezoelectric transducer driven system is regarded as a Spring-Mass-Damping (SMD) system (see Figure 7). As shown in Figure 8, the EMI method measures the electrical parameters of piezoelectric transducer mounted on or embedded into its host structure. The transducers used in EMI method work as both actuators and sensors.

#### 2.2.3. Impact Monitoring

**Figure 9:**
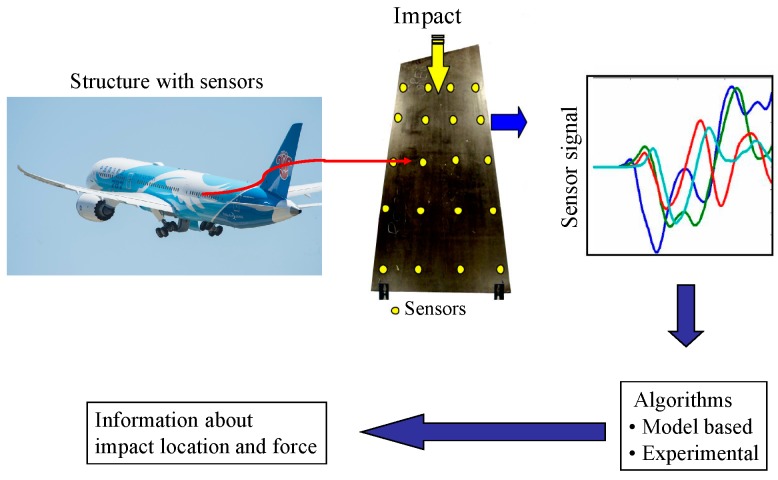
Principle of impact monitoring.

The passive diagnostic hardware consisting of sensor amplifier and data acquisition unit is simpler than the active diagnostic hardware. The function of impact monitoring system is to collect stress wave signals generated by impact loads through sensor network on the structure, estimate impact location and reconstruct impact energy or force history, so as to monitor external impact on aircraft structure. The principle of impact monitoring is shown in Figure 9.

#### 2.2.4. Integrated Passive-Active Monitoring

Purely passive systems exist only for detecting impacts and other load changes, with any diagnosis of damage or further analysis being left to other systems. In contrast, purely active systems used to detect damage also suffer from their own drawbacks. For instance, active systems must constantly query the structure, resulting in wasted energy and expense when they are not needed. Therefore, it is desirable to combine active and passive systems in order to bring forth the advantages of both systems. With the active-passive diagnostic hardware integrated within a single sensor layer, upon detection of impact event, the transducers are engaged in an active mode to actively scan the impact area to determine the location and size of any resulting damage region. Details of the active-passive diagnostic system can be found in the literatures [33,34].

## 3. Identification Algorithms of Damage and Impact

### 3.1. Algorithms for Wave Propagation-Based Damage Detection

Algorithms for damage detection using guided wave were extensively investigated during the past two decades. Comprehensive reviews on the state of the art of Lamb wave-based damage identification approaches can be found in literatures [35,36]. The unique characteristics and mechanisms of Lamb waves in laminated composites, approaches in wave mode selection, generation and collection, modelling and numerical simulation techniques, signal processing and identification algorithms were well summarized in literature [37]. There are various processing techniques of Lamb wave signals, including time-series analysis [38], frequency analysis [39] and integrated time–frequency analysis [40]. Algorithms for Lamb wave-based damage identification can be either forward or inverse [41,42,43]. Logical analysis of signals may be sufficient for few simple cases, while quantitative damage identification can only be effectively achieved through appropriate inverse algorithms. Finite element techniques have been widely used for understanding of wave characteristics, and validating signal processing. Comparing with finite element method (FEM), spectral finite element method (SFEM), provides an exponential rate of convergence. It is a more promising tool in the investigation of lamb waves propagation in plate like structures [44,45]. Frequency domain spectral finite element (FSFE) method is another widely used semi-analytical technique for guided wave propagation simulation [46]. Hybrid modelling technique for efficient simulation has also been developed [47]. Shen and Giurgiutiu developed the combined analytical FEM approach (CAFA) to include damage effects in the 2-D Lamb wave analytical model [48].

In recent years, damage diagnosis imaging methods based on ultrasonic guided waves have been widely concerned and developed to depict the position and area of damage. The main damage imaging methods include phased array imaging, delay and sum, tomography, migration imaging, and so on. The comparisons of several damage detection techniques are listed in Table 3.

In phased array imaging, several PZTs in the sensor network are used to form a dense sensor array. It is the same as the principle of a radar that the phase of excitation signal of each PZT can be adjusted independently so that Lamb wave propagation can be focused on the specific direction for the far field or on the specific location for the near field because of the sum of Lamb wave field excited by all PZTs. If the guided wave beam is controlled to focus to every corner, omnidirectional scanning and rapid detection of a plate-type structure with limited access can be realized. Giurgiutiu et al. [43] realized damage detection of guided wave-based phased array technology by using a linear PZT array. PZT array configurations like circular, rectangle, cross-type were studied and optimized to reduce the monitoring blind area, suppress the sidelobes, real the omnidirectional scanning [49,50,51,52,53,54]. Some researchers employed multiple arrays for data fusing to improve the accuracy of damage diagnosis results [55,56,57]. Phased array damage imaging for anisotropic composites was also investigated [58,59,60,61]. From the investigation, it was found that it is difficult to identify the damage location in accuracy for all the directions. Yu et al. [62] pointed out that the energy skew between the direction of group velocity and phase velocity must be considered for anisotropic material and proposed a general phased array algorithm.

In the Delay-and-Sum (DAS) method, according to the damage scattering signal received by multiple pitch-catch paths, the damage is located according to the group velocity and the propagation time of guided wave. The scattering signal is processed by short-time Fourier transform, wavelet transform or Hilbert transform. The signal energy after signal processing is mapped to the potential damage point, which results in that the signal energy summed at the location of real damage reaches a maximum. DAS imaging formulation was firstly proposed and verified by the experiment by Wang et al. [63]. Michaels et al. [64,65] further studied DAS imaging method and improved imaging performance by the exponential window and fusing multiple-frequency signals. They also proposed a variation of DAS imaging algorithm, named minimum variance distortion-less response imaging method, where weight coefficients are adaptively calculated at each point [66]. Qiu et al. [67] proposed a DAS-based multi-damage monitoring method, which divided the monitoring area into multiple subarea and proposed a damage index merging algorithm to fuse the signals from adjacent subareas. They proposed the concept of multiple subareas monitoring to divide the whole monitoring area into small subareas, and DIMA was introduced for quick damage identification in every subarea and obtaining the damage number. Each damage was localized independently only with the information in its own merging subarea. For damages locating on or near the common channel of two neighboring subareas, two subareas were treated as one merging subarea, and their information was utilized together for damage localization. For anisotropic composites, the correlation between group velocity of guided wave and propagation angle should be taken into account to real an effective damage imaging [68,69].

Ultrasound guided wave tomography (GWT) originates from the X-ray computer tomography (CT) technology commonly used in medicine. In this method, sensors are usually arranged around the monitored area and form a great number of transmitting-receiving paths. The monitoring principle is that the signal changes of guided wave varying with the damage are fusing to contour the damage. Common methods of guided wave tomography include standard parallel projection, fan-beam projection and double-cross projection [70]. Khare et al. [71] and Balasubramaniam et al. [72] found that cross projection having the strongest adaptability, is suitable for scanning any shape and can improve the tomography effect of anisotropic composites. In addition, the diffraction tomography method was proved to be more suitable for damage imaging of anisotropic composites than the linear ray tomography method [73,74]. Rose et al. [75] proposed a simple correlation coefficient-based tomography method, named Reconstruction Algorithm for Probabilistic Inspection of Damage (RAPID), which might reduce the computation cost for an accurate monitoring result. Based on RAPID algorithm, Wang et al. proposed a damage index based on information entropy to emphasize the global difference and eliminate the temperature effect on guided wave [76]. Wang et al. also proposed a virtual sensing path method to increase the number of sensing paths to improve the imaging quantity [77]. Hua et al. [78] combined the advantage of both RAPID and DAS algorithm and proposed a local signal difference coefficient to improve the accuracy of damage detection. Liu et al. [79] modified the weight coefficients of RAPID to make the algorithm more suitable for the case with few paths.

Gao et al. [80] proposed a three-step method based on RAPID to visualize the multi-damage in the complex structures. The multi-damage diagnosis strategy includes a path damage judgment stage, multi-damage judgment stage and multi-damage imaging stage. The multi-damage identification method was employed to inspect multi-damage in an aluminum specimen. The result showed that the presented guide waves-based multi-damage identification method is capable of visualizing structural multi-damage.

Most of wave propagation-based damage detection methodologies rely on the use of baseline data collected from the structure in the undamaged state. It is well known that some environmental effects, such as temperature, load, humidity, radiation, will also cause changes in the sensor signals, and will thus interfere with most damage detection schemes and cause the false alert. There are some research reports about temperature compensation to reduce the effect of the change of amplitude, phase and wave shape, such as baseline signal stretch (BSS) [81], Optimal baseline subtraction (OBS) [82], combination of BSS and OBS [83,84], and combination of adaptive filter and optimal baseline selection [85]. With the novel temperature compensation technique combining an adaptive filter and optimal baseline selection to enhance the robustness and effectiveness of guided wave-based damage detection, it only requires a few baselines for a large temperature range. Experimental results showed that temperature interval for baselines of low frequency signals, such as 50 kHz, could be up to 20 °C [85]. There are also some studies about load compensation, which focus on reduce the load effect on guided waves about three points: the structure deformation caused by load [86], acoustoelasticity due to the present of stress [87] and the load influence on piezoelectric constants [88]. Guided-wave based SHM system was also investigated for localization of barely visible impact damage in CFRP plates under vibration and different thermal conditions. Barely visible impact damage was localized on a CFRP plate with a single pristine baseline recorded at 25 °C in a temperatures range of −50 °C to 60 °C by applying temperature compensation [89].

To overcome the limitations of environmental effects, baseline free damage detection technique was proposed. Baseline free time reversal method has been extensively studied by the researchers [90,91,92,93,94]. Wang et al. [90] demonstrated its use to detect damage. Sohn et al. [91] illustrated time reversal process of Lamb waves for damage detection by examining the deflection of the reconstructed signal from the original signal in a composite plate containing delamination damage. Poddar et al. [92] used time reversal method of Lamb wave for the detection of notch, block mass and corrosion surface type of defects in a metallic plate. Bijudas et al. [93] did the similar study on composite laminate and T-pull composite specimen. Baseline free methods have also been employed to identify, locate and quantify the damage in the composite structures under varying temperature conditions without the comparison of Lamb wave signals obtained from the best condition of the targeted structure [94]. However, the real world application of baseline free damage detection technique is difficult because of the complex aircraft structures. Damage signals can be scattered and mixed with the signals coming from the wave source and those which are reflected from the boundaries of structure.

In addition, a novel methodology for optimal sensor placement based on maximum area coverage for damage detection using Lamb waves was also developed. A feature of the optimization approach lies in the fact that it is independent of characteristics of the damage detection algorithm making it readily up-scalable to large complex composite structures such as aircraft stiffened composite panel [95].

### 3.2. Algorithms for EMI-Based Damage Detection

The comprehensive reviews on EMI methods can be found in the literature [24,96,97,98]. This paper focuses on the issues on how to apply the EMI methods on aerospace engineering in real world, especially for composite structures. These issues include damage characterization, environmental effect compensation, lightweight sensor design and installation.

The EMI methods have been demonstrated to effectively detect various damages, such as crack and load damage [99,100], debonding of composite adhesive layer [101,102], corrosion of metallic structures [103], bolt loosening of mechanical joints [104]. Damage is typically characterized using damage indices (DIs) that are based on the comparison between two electrical impedance signatures (either real part or imaginary part, or their modules), where one of them is acquired when the structure is in a healthy state. Generally, several kinds of DIs are defined by statistical equations and widely used for quantitative assessment of EMI signatures [95,105,106]. These statistical DIs include the root mean square deviation (RMSD) [107] and mean absolute percentage deviation (MAPD) [108], both of which are suitable for locating and characterizing damage; covariance (Cov) and correlation coefficient (CC) [109], which can be used to identify damage size at a fixed location; chessboard distance (CBD) [110], which can be used to investigate the EMI signatures varying with temperature; average square deviation (ASD) [111], which can be used to effectively locate damages via the degree of signatures dispersion from healthy state; united mechanical impedance (UMI) [112], which is able to reflect the changes in structural properties caused by damages; ellipse damage index (EDI) [113], which is able to be used for damages detection inside the structures in a very initial state.

It should be noted that the affectivity of DIs depends on the selection of EMI signature components (the real and imaginary part) and the way of EMI signature measurement and conversation. For example, Budoya and Baptista [25] used two measurements, i.e., transient-state measurement using a sweep excitation signal for all the components of the desired frequency range, and steady-state measurement using a pure sinusoidal signal for each frequency of interest. The results showed that the steady-state measurement using RMSD was more precise and sensitive to damage.

To quantitatively identify the type and size of damage, lots of work were further investigated. Since the EMI signals are usually obtained in the frequency domain, it is not easy or intuitive to make implementation. Therefore, some researchers were making attempts to analyze the EMI signals in a view of time domain [114,115,116]. An interesting and noteworthy work was done by Zahedi and Huang (2017) [116]. Through inverse fast Fourier transform and Hilbert-Huang transform, the EMI signature, essentially a pulse-echo signal represented in the frequency domain, could be converted to a time-domain pulse-echo signal at any given excitation, where Resonant phase and Echo phase were used to identify the damage type and the location, respectively. The experimental results showed that the method could perform physical-based damage detection and characterization.

Similar to guided wave signals, the EMI of piezoelectric transducer can be significantly affected by environmental and operational conditions, such as loads, vibration and temperature [117,118], as well as the thickness of adhesive layer between PZT and the host structures [119]. Research conducted by Wandowski et al. [120] demonstrated two common ways to eliminate the influence of environmental factor fluctuation. One was to select a suitable DI to minimize the signature variations due to condition change, for example, DIs based on cross-correlation. The other was to develop effective compensation approaches. Generally, these compensation methods were based on advanced signal processing tools integrating with artificial intelligent algorithms such as Artificial Neural Network (ANN), genetic algorithm, and fuzzy reasoning [121]. A SHM strategy integrating the EMI method with the Lamb wave method for composite pressure vessels under varying internal pressure loading environments was proposed by Gao et al. [122]. A new damage index adjusting Lamb wave damage detection was developed based on the features of real and imaginary parts of the EMI signals. Although some techniques have been presented to compensate the environmental effects of EMI methods, it is still a challenge to compensate or eliminate these effects completely.

The piezoelectric transducer is required to be small and lightweight enough when EMI method is used in aerospace application. Current transducers are often designed using piezoceramics, so-gel thin-film, composite piezoelectric films. The design and manufacture aspects of piezoelectric film deposition on different substrates are an interesting hotspot. For example, Hoshyarmanesh and Abbasi [123] deposited the integrated composite piezoelectric films on the blades of turbo-machine prototype. They developed a wireless data measurement system and investigated the influence of different temperatures and rotational speed on the EMI signatures. The EMI signals showed a clear frequency shift of existing peaks and the appearance of new peaks when the damage increased to a secure minimal detectable size. It demonstrated the feasibility of the proposed method for incipient damage detection on rotary structures prior to any failure. Na and Lee [124] developed a method to avoid attaching the piezoelectric transducer directly onto the target structure using a metal wire for damage detection at high temperature. By shifting the frequency to compensate the signature changes subjected to the variations in temperature, their experimental results indicated that damage identification was successful above 200 °C, making the metal wire method suitable for the EMI technique in high temperature environments.

### 3.3. Algorithms for Impact Monitoring

Many studies have been conducted for impact identification, including estimation of impact location and reconstruction of impact force history or energy [125,126,127,128,129,130,131,132,133,134,135,136,137,138,139,140,141,142,143,144,145,146,147,148,149,150,151,152,153,154]. There are two basic approaches for impact identification: one is the model-based technique, the other is the neural network-based technique. The model-based technique is based on mathematical models which can describe the dynamic characteristics of stress wave propagation in a structure. Due to the complex system characteristics and unknown boundary conditions, most model-based studies were performed primarily on simple structures and they are not easily applied to real complex structures in practical applications.

Gaul et al. presented a method that uses piezoelectric films to detect the flexural waves in plates [125]. The arrival time, velocity and distance of the wave are determined at different frequencies, and used to identify the impact location with optimization algorithm. On the basis of Gaul’s work, Meo et al. proposed an impact detection algorithm to locate the exact position on an orthotropic plate [126]. This algorithm used time lag between impact and sensors as well as the trigonometric identity to solve the inverse problem to locate the impact. Zhang et al. developed an imaging method based on probability calculation for locating impact on a plate-like structure [127].

**Figure 10:**
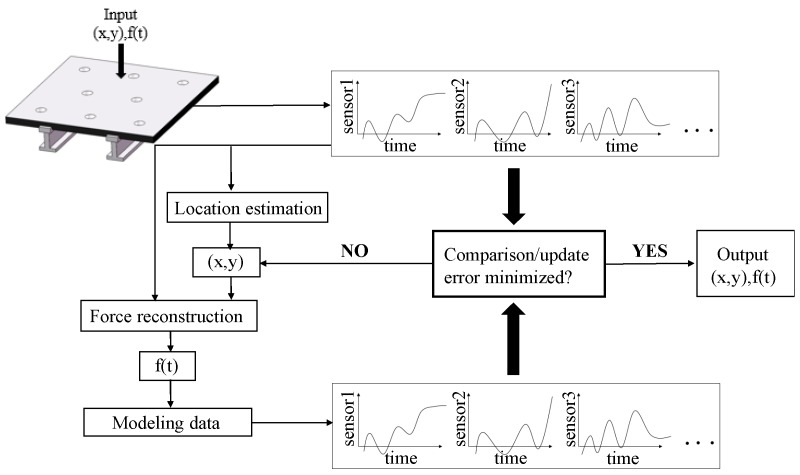
The principle of model-based methods.

Choi et al. proposed an optimal smoothing and filtering algorithm based on distributed built-in piezoelectric sensors to reconstruct the force history of impact on composite beams [128]. This impact identification algorithm solved the inverse problem by using system model and a response comparator. The system model described the relationship between input (external force) and output (sensor signals). Response comparator compared the measured sensor output signal with the predicted result of the model, and adopted the filtering and smoothing algorithm to update the impact location and the force history of impact. Tracy et al. extended the application of this algorithm on beams to the plate structures, and developed the computer code, IDIMPACT [129]. For dynamic problems, local changes in properties has a huge effect on the response of the structure. Therefore, in the modeling, the local effect of regional variation in structure properties due to the presence of stiffeners must be taken into account. Seydel et al. conducted an investigation to develop an impact identification system for the prediction of force location and history of low-velocity impact on the stiffened plate [130,131]. When the impact occurs, the system approximately estimates the impact location at first, and then reconstructs the force time history. The actual impact position is then updated by minimizing the difference between the model response data and the actual response data through the iteration process. The principle of model-based method is shown in Figure 10 [130].

Park et al. further developed the impact identification algorithm using a built-in diagnostic technique. This method does not rely on the full-scale mathematical model of structure, nor does it require large scale acquisition of training data to establish neural network, but obtains the transfer function which can reproduce the physical behavior of system through experimental testing [132]. Mueller et al. improved the method proposed by Park, and presented a two-step model calibration method, which could simulate the impulse response in the training process of ARX model to reduce the simulation error of the numerical model in the early window and improve the accuracy [133]. Ahmari and Yang proposed a simplified method for detection of impact localization and energy of simple support plates based on finite uncertainty measurement [134,135]. This method can find the maximum deviation range of impact location and energy on the plate.

It should be pointed out that the model-based method is quite intractable for complex structures. Even if it can simulate the response well, it is difficult to reconstruct the excitation formula of input force from the output sensor signals simply.

Worden and Staszewski developed an algorithm for impact location and quantification on a composite panel using neural networks [136,137,138]. They also investigated the problem of optimal sensor distribution for impact detection and localization on composite materials. LeClerc et al. improved the method, combined the classifier and the regression algorithm based on the two-step method and used the similar features of simple structures to obtain the impact location on complex structures [139]. According to this method, the complex structures is divided into several sub-regions. When the impact occurs, first, the classifier is used to define the sub-region where impact occurs, and then the impact location in the sub-region is refined by regression network. Chen and Yuan [140] investigated impact source identification in finite isotropic plates using a time-reversal method. Marchi et al. [142] developed a passive monitoring technique based on dispersion compensation to locate impacts in plate-like structures. Kunda et al. [143] proposed a technique to locate the acoustic source in large anisotropic plates with the help of only six sensors without knowing the direction dependent velocity profile in the plate.

Recently, Fu et al. [145] applied Kernel extreme learning machine (kernel ELM) to predict the location of impact event on a clamped aluminum plate that simulates the shell of aerospace structures. Merlo et al. [146] proposed a novel Differential Time-of-Arrival (DToA) estimation technique for improving the accuracy of impact position determination using acoustic source triangulation schemes based on the data collected by piezoelectric sensors attached to the structure. Zhu and Qing proposed a two-step impact localization method for composite structures with a parameterized laminate model [147], The method is divided into two steps: one is to inverse material parameters by two impacts with known locations, which is called the calibration step. The other is to inverse impact locations when the material parameters are known, which is called the monitoring step. The arrival time of flexural waves extracted by continuous wavelet transforms is used as input features during the inverse process. Khodaei et al. [148] established a metamodel, with the help of an ANN, capable of estimating the coordinates of an impact from sensor readings. Using the established ANN, the locations of impacts of different energies were successfully detected over a full composite stiffened panel.

It is well known that wireless sensor networks (WSNs) offer numerous advantages over conventional wired systems, such as low weight and cost, scalability, flexibility, and ease of deployment. Some studies using a piezoelectric transducer based WSN to detect impact for aerospace and other industries have been conducted [149,150,151,152,153]. In order to meet the needs to monitor impact on line for large scale aircraft structures with low weight and low profile requested, Yuan et al. proposed a novel multi-response based wireless impact monitoring network which can unite multiple leaf nodes to solve the problems of localization confliction and mid-region localization. The hardware architecture of wireless impact monitoring network developed by Yuan et al. can be found in literature [154]. Fu et al. [155] designed an innovative low-power high-response wireless structural health monitoring system for impact detection of composite airframes. In order to effectively monitor the rare, random and transitory impacts on aircraft structures, an event-triggered mechanism was adopted for the system to exhibit low power consumption when no impact occurs and high performance when triggered.

## 4. Requirements for Practical Implementation of SHM

### 4.1. Design Principles of SHM System

To design an integrated SHM system that can be practically used for aircraft applications in real world, some main issues shall be considered carefully: the definition of system function, composition of the system, and airworthiness compliance, and so on.

#### 4.1.1. Definition of System Function

The objectives to be monitored for aircraft application, especially for civil aircraft, can be classified to two categories: one is monitoring of structure state parameters, such as impact energy, strain, temperature; the other is detection of structural damages, such as cracks, delaminations, corrosion, and so on. Before an SHM system is designed, the functions of the SHM system should be well defined for the specific application based on what is required to be monitored and the readiness of the technology used to monitor it. In general, impact damage over large areas in composite structures, cracks at hot spot areas and strain distributions at some critical areas must be monitored.

#### 4.1.2. Composition of SHM systems

**Figure 11:**
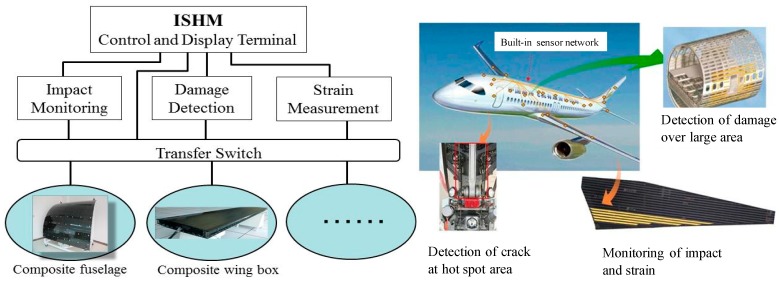
Architecture of integrated SHM.

As described above, there are many types of sensors that can be used for SHM, including piezoelectric, fiber-optic, ICM, CVM, MEMS, strain gauges, etc. Besides piezoelectric transducers, it is often necessary to integrate various types of sensors in an integrated SHM system to complete the multidimensional measurements of structure since each individual sensor has its own advantages and limitations. For the giving conditions of usage and structural application, the sensor technology selected for the integrated SHM system shall meet three criteria: (1) the function to complete a specific measurement, (2) the capability to be integrated with the host structure, and with other hardware systems to constitute integrated SHM system, and (3) the maturity and network capability. Figure 11 provides the brief architecture of integrated SHM in aerospace applications.

#### 4.1.3. Airworthiness Compliance

The SHM system used for aircraft structures must meet the requirements of airworthiness compliance. According to the characterization of SHM system, its airworthiness compliance needs to be considered in three aspects:

(1) As an electric device on the aircraft, the SHM system must meet the electronic and electrical regulations for airworthiness, which include requirements about power supply, anti-static protection, fireproofing, high-intensity radiated fields (HIRF) protection, lightning protection, and anti-burst of oil tank, etc.
(2) The integrated SHM system shall meet electrical wiring interconnection system (EWIS) regulations. The design and installation of wires, cables, connectors and switching devices shall satisfy with the requirements of electrical grounding, physical isolation, line shield and protection, and material flame-retardant, etc.
(3) As an installed structure on the aircraft, the sensor network of SHM system shall meet the installation regulations for airworthiness, which include the requirements concerning weight, load distribution, material compatibility of sensor and adhesive layer with the aircraft structure, applicability and durability of sensor network, accessibility and detectability of the overall system, etc.

More specifically, the following requirements shall be addressed for designing an SHM system in an aircraft application: (1) function and performance requirements, including the capabilities of detecting and assessing structural damage, the minimum damage size to be detected, probability of detection (POD); (2) operation requirements, including ease for setup, a simple GUI; (3) safety requirements, including no adverse affect on the structural integrity of aircraft and personnel using it; (4) reliability & environmental requirements; (5) installation requirements; (6) maintainability requirements; (7) weight requirements.

### 4.2. Some Key Issues for Implementation of SHM System

Besides the airworthiness compliance described above, there are several key issues that need to be addressed before the SHM technology can be widely used for aircraft. These key issues include: capability of large sensor network, self-diagnosis, environmental adaptability and quantitative monitoring results.

#### 4.2.1. Capability of Large Sensor Network

Aircraft structures to be monitored are typically large in size, and need a large sensor network for sufficient damage detection and structural state sensing. Building such a multifunctional large sensor network for aircraft structure is very difficult. There are many challenges and issues that need to be solved, including: (1) how to build a large sensor network and integrate a variety of sensors together, (2) how to establish the communication link between the sensor networks and processing center, (3) how to process all the information from a variety of sensors. SMART Layer technology provides an effective way to integrate a certain number of sensors and actuators with metal/composite structures [15]. However, as the number of sensors increases, the integration of such a network with a large structure using SMART Layer still can be very challenging or become impractical.

**Figure 12:**
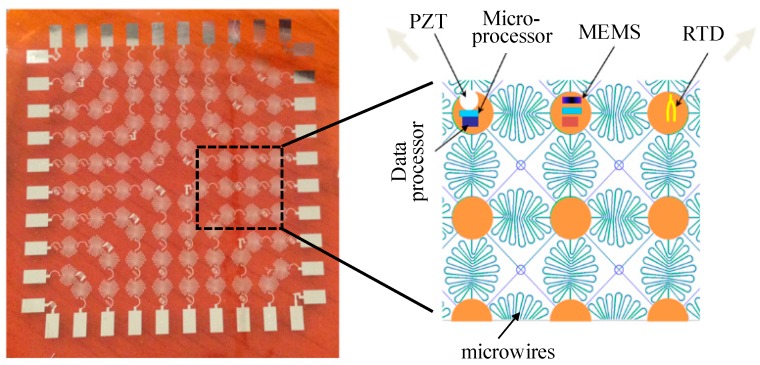
Expandable multifunctional sensor network.

The concept of multi-modal sensing capabilities with built-in sensor network was proposed. An expandable lightweight polymer-based flexible sensor network that can host a high-density array of multiple types of sensors was developed [156,157]. As shown in Figure 12, the flexible sensor network can span large macroscopic areas and can easily be integrated in composites with negligible impact on the composites mechanical performance.

#### 4.2.2. Environmental Adaptability

The SHM system designed for aircraft applications in the real world needs to provide the defined functions under different operation environments of aircraft. Survivability of the SHM system and reliable results under these environments are two important issues of environmental adaptability.

The durability of the PZT sensors must be entirely guaranteed through the life of the host structure. However, the strength of the PZT is often much lower than that of the host structure, which is a critical problem to be solved urgently. It was demonstrated that the PZT bonded the structure can be used without any degradation even if the applied strain to the host structure exceeds the failure strain of PZT by introducing compressive pre-stress via a specific cure cycle [158]. Another novel approach to greatly improve the strain survivability of PZT by optimal design of adhesive used to bond them to the host structure was proposed recently by Sun and Qing [159]. Based on the theoretical model established, finite element method developed and experiments conducted, it is clear that the PZT sensor can work perfectly when it is mounted on the host structure with tensile strain up to 4000 με by using optimal adhesive, such as Hysol EA 9395 with an adhesive thickness of 125 μm. This is because the strain transferred to the PZT is still less than 1100 με, which is the maximum tensile strain PZT can bear, even the strain of host structure reaches 4000 με.

The reliability of piezoelectric transducer based SHM system under harsh environments has been investigated [20,22,160,161]. Research results demonstrated that the sensor network can work well over a large temperature range, from the temperature of liquid nitrogen [21] to the cure temperature of carbon fiber composite materials [161].

#### 4.2.3. Self-Diagnosis

If one or more sensors are degraded, damaged, or missing, the SHM system may not function properly and can give false indications of structural damage. Measuring the impedance of each channel can be used to find an open or short circuit. This can indicate a missing sensor or damaged connection/wiring, but a degraded or damaged sensor may go undetected using the impedance method. To resolve this problem, a reasoning process using the active sensor signals has been developed and implemented to detect degraded or damaged sensors that the impedance method may miss [160].

**Figure 13:**
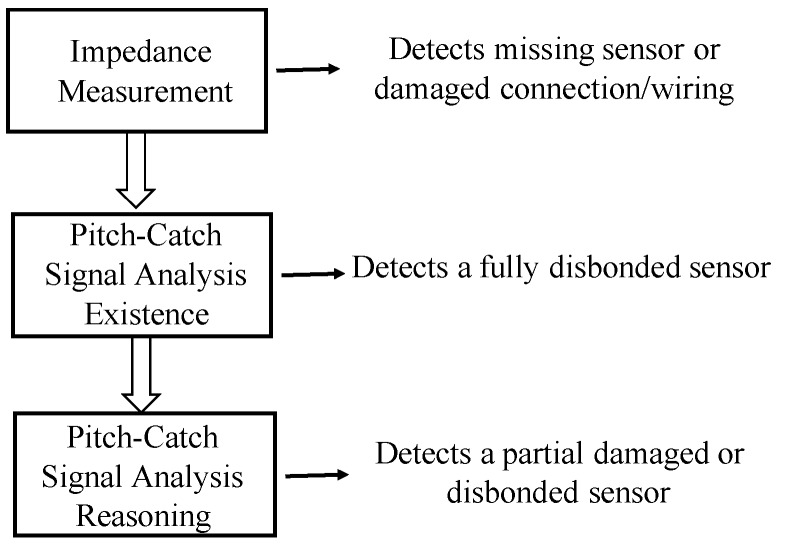
Integrated three-step method to automatically detect faulty sensors.

An integrated three-step method shown in Figure 13 has been developed to automatically detect faulty sensors caused by a missing sensor or damaged connection/wiring; a sensor that is still connected to the electronics, but is disbonded from the structure; and a partially damaged or disbonded sensor. If a sensor is flagged as degraded, damaged, or missing, all signal data from the faulty sensor are removed from the analysis routines.

#### 4.2.4. Quantitative Monitoring Results

One of the elements that are essential to the practical usage and implementation of any structural health monitoring system for aerospace applications is to provide well-defined resolution including a quantifiable probability of detection (POD) and damage size/severity along with measure of uncertainty when a damage is detected. Although many SHM systems have demonstrated that they can detect and locate damage on a structure, providing quantified damage sizes still remains a challenging task, even in a laboratory environment.

For the detection of impact damage over large area in composite structures, probability of detection (POD) was introduced as a standard measurement for quantifying the reliability and robustness of built-in structural health monitoring systems. However, traditional POD curves are generated through extensive testing which is not practical for every new structure and sensor configuration. To overcome this difficulty, Acellent has developed a method to compute the POD [14,162]. After a sensor layout has been defined and the operating paths have been determined, the user can select the option to generate POD curves. The computations use the geometry of the sensor configuration and the actuator-sensor paths, along with the logic in the damage detection reasoning algorithm, to generate the POD curves.

**Figure 14:**
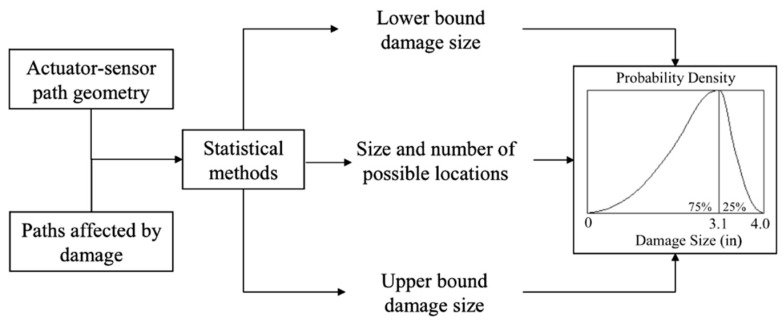
Probable damage sizing using statistical methods.

Qing and Beard et al. [163] proposed a probability density curve for the damage size determination, giving lower and upper bounds on the size, as well as the most probable size, as shown in Figure 14. In addition to the most probable damage size, the percentage chance that it is smaller or larger than the most probable size is computed by the area under the curve. The damage is assumed to be a circle and the calculations for the damage size are based on the geometry of the actuator-sensor path.

There are also a lot of studies devoted to making use of statistical and machine learning methods to solve the uncertainty problem caused by environmental variation, dispersive effect, measurement error, and so on. The use of an artificial neural network (ANN) was proposed for Lamb wave-based damage detection in laminates by Su et al. [164]. Dworakowski et al. [165,166] developed damage index-based ANN for complex structures.

Support vector machine (SVM) with robust anti-noise characteristics was employed for damage classification with more detection rate than ANN by Agarwal et al. [167]. Some classical statistical algorithms, such as principal component analysis [168] and maximum-likelihood estimation [169], have been also utilized for damage detection. Bayesian approach was proposed by Ng [170] for damage identification in beam-like structures and then developed for damage imaging in plate-type structures by Yan et al. [171,172]. The Gaussian mixture model was firstly proposed by Chakraborty et al. [173] for Lamb wave-based crack propagation tracking and further developed by Yuan’s group to realize on-line updating of fatigue crack tracking [174,175] and path-synthesis accumulation imaging [176]. A particle filter method was also utilized for damage location by Yan [177] and fatigue crack prediction by Yuan’s group [178]. There are also some challenges for guided wave-based statistical and machine learning methods, which have the deficiency of requiring training data, the existence of outliers and so on. Some other algorithms may improve the performance of statistical and machine learning method, such as deep learning, boosting, semi-supervised learning, etc.

## 5. Development Trends of Structural Health Monitoring Technology

After more than 20 years of development, SHM technology is being used more and more in the aerospace industry. The objects of SHM application have gradually expanded from simple metallic structures to more complex composite structures, while the monitored physical parameters have expanded from strain and temperature to various kinds of damage, and the diagnostic results have gradually developed from qualitative to quantitative. Focusing on the form diversity, feature complexity, damage diversity and concealment of aircraft structures, exploring the development trend of health monitoring of aircraft structures from monitoring process, innovative sensor design, new monitoring methods or technologies is an important way to improve its technical maturity. In the field of aerospace industry, the development trends of SHM can be summarized as the following [179,180]:

(1) The sensing technology is developing to the multi-field coupling sensing technology.

Sensors have been widely applied on aircrafts to monitor the strain, temperature, etc., of the aircraft structure, but the damage detection SHM technology can bring direct benefit to the maintenance of aircraft, which is still in the laboratory and flight testing stage, and also only focuses on small local areas. From relatively simple monitoring of stress/strain, temperature to direct monitoring of damage is an obvious development trend of SHM [181]. In the near future, SHM technologies will be developed from a single parameter monitoring of the local area with a few sensors to multi-parameter monitoring of the large structure with large sensor networks.

(2) Sensors are developing towards miniaturization, intellectualization and in-situ integration, and then can be seamlessly integrated with composite structures.

With the rapid development of materials science, manufacturing technology, microelectronics and information science, many multi-functional materials have emerged, which can integrate sensing, actuating, communicating and computing. They provide a technical basis for the miniaturization, integration and intelligent development of sensors [182]. It is an important direction to develop composite fibers or matrix materials with sensing function by nanotechnology to make the composite structure itself be a functional sensing system. For example, carbon-sodium nanotubes can be added to composite structures to form in-situ sensors, and then structural strain, humidity and temperature can be monitored by resistance changes [183].

(3) The monitoring process is developing to the life cycle of structure design, manufacture, service and maintenance of aircraft structure, especially aircraft composite structure.

Composite materials have been and continue to be widely used in aerospace because of their high strength-to-weight and stiffness-to-weight ratios. Due to the complexity of aircraft composite structures and the harsh service environment, the damage and failure mechanisms of composite structures are diverse. It is difficult to fully characterize the service state of composite structure by a single physical parameter. The development of multi-field coupled hybrid sensor networks and combinatorial characterization methods are necessary. The construction of the multi-mode holographic sensing capability, the realization of health monitoring of composite structure from design, manufacture, service to maintenance of life cycle, and the formation of innovative concepts and design methods of smart composites are the important development direction of SHM.

(4) Monitoring methods are developing from linearity to nonlinearity, from low frequency to high frequency, while diagnosis results are developing from qualitative to quantitative.

With the development of solid mechanics, computational mechanics and non-linear theory, non-linear monitoring methods (such as non-linear ultrasound [184], non-linear guided wave [185,186], and non-linear solitary waves [187]) have shown advantages in micro-damage identification, and characterization. The frequency of excitation source required increases correspondingly and extends to high frequency region. This is the current and future focus of structural health monitoring academia. The main characteristics of high-frequency non-linear monitoring method are more precise calculation and large amount of data.

From the application point of view, the academia and industry are also paying more and more attention on artificial intelligence technology and statistical methods to process a large number of data in order to obtain more reliable and accurate quantitative diagnosis results. In addition, with the increase of monitoring parameters and the diversification of data types, the SHM technology in large data environment has attracted increasing attention from academia and industry. It is expected that the quantitative diagnosis results will be input into the decision-making process of aircraft life prediction and condition-based maintenance, and feedback to the design process.

(5) The performance of SHM systems is developing from detection of event, location and size of damage to the detection of effects.

In order to quantify the performance of SHM systems, we can classify them by their output with four sequential levels called Technology Classification Levels (TCL): Detection of event (TCL1), Detection of location (TCL2), Detection of magnitude/size (TCL3), Detection of the effect (TCL 4) [14]. At present, most studies are focused on sensors, systems, and diagnostic algorithms to output monitoring results (TCL1, TCL2, TCL3). From the general concept of aircraft health management, it is necessary to input the monitoring results into the residual strength or residual life model of the structure to evaluate the effect of damage (TCL4), in order to truly realize the durability of damage tolerance design, and achieve the benefits of structural weight reduction and maintenance cost reduction. A multi-source life prediction model based on physical model (such as continuous damage mechanics and fracture mechanics model), system identification and data-driven method can be constructed to predict the life and performance of the aircraft structure by means of cooperative analysis technology based on monitoring and diagnosis results and state prediction model. In addition, Khodaei et al. [188] proposed a multi-level decision fusion strategy which weighs the value of information against the intended functions of a SHM system. The three different intended functions of SHM include: Level 1—damage existence, Level 2—damage detection and approximate location, and Level 3—damage detection and identification. The advantage of strategy is that it is readily scalable for large complex structures.

## 6. Summary

Structural health monitoring will have a great impact on structure design, improving safety and reliability, and reducing maintenance and operation costs for aircraft. Among the various types of transducers, piezoelectric materials are being widely used for SHM because they can be used as either actuators or sensors.

An overview of piezoelectric transducer-based SHM technology developed in the past two decades was first provided in the paper. SMART Layer-based sensor networks, the principles of active and passive monitoring, and diagnostic algorithms were briefly summarized.

The requirements for practical implementation and use of SHM systems in aircraft application, and the challenges needed to be solved in system integration, damage quantification, environmental compensation approach, and airworthiness compliance approach in order that the SHM technology can be widely used in aircraft, were then addressed.

Development trends of SHM technology were also discussed in this paper. The object of SHM applications has gradually expanded from simple metallic structures to more complex composite structures, while the the monitoring of physical parameters has expanded from strain and temperature to various kinds of damage, and the diagnostic results have gradually developed from qualitative to quantitative.

Although extensive studies are still needed in the areas of damage quantification, environmental compensation, system integration, airworthiness compliance and verification method to fully implement an SHM system in aircraft applications, it is clear that the SHM technology will provide a cost-effective, in-service, cradle-to-grave diagnostic system for monitoring the structural integrity of aircraft.

## Figures and Tables

**Table 1:** Comparison of traditional NDT and SHM technologies.

Key Features	Traditional NDT Technology	SHM Technology
Transducers	Be separated from the structures or mounted on the structures temporally	Be mounted on or embedded into structures permanently
Detection mode	Off-line	Off-line and on-line
Inaccessible region	Not be inspected in service	Be monitored in service
Downtime	Be increased due to schedule detection	Be reduced through real time monitoring
Detection time	Time-consuming	Automatically and quickly obtain information
Detection capacity	Just provide flaw information	Sense structure states, including flaw, strain, temperature, et al.

**Table 2:** SHM technologies commonly used in aircraft.

Monitoring Principle		Sensor	Monitoring Object	Mode
Strain		Fiber optical sensor	Loads and impact	Passive
Wave propagation	Stress wave	Piezoelectric sensor (e.g., PZT, PVDF)	Impact	Passive
	Acoustic emission	Piezoelectric sensor	Global /local damage	Passive
	Guided waves	Piezoelectric/electro-magnetic sensor	Global /local damage	Active
	Ultrasonics	Piezoelectric sensor/Laser	Local damage	Active
Electro-mechanical impedance		Piezoelectric sensor	Local damage	Active
Electric resistance		Resistance element	Local damage	Passive
Intelligent coating monitoring		Nano material	Local damage	Passive
Comparative vacuum monitoring		Air/vacuum galleries	Local damage	Passive
Eddy current		Eddy current foil sensors	Local damage	Active

**Table 3:** Comparisons of several damage detection techniques for SHM.

Algorithm	Superiority	Shortcoming	Sensor Density	Capability of Engineering Application
Phased array [42,48,49,50,51,52,53,54,55,56,57,58,59,60,61]	High accuracy	Must identify the wave mode and its group velocity	Compact array	Simple plates
Delay and sum [62,63,64,65,66,67,68]	Simple algorithm; Capability of multi-damage	Must identify the wave mode and its group velocity	Sparse	Large-area plate-type structures
Tomography [69,70,71,72,73,74,75,76,77,78,79]	Do need to identify the wave mode; Simple algorithm	Need a great number of paths	High density	Large-area complex structures
